# Supplementary figures and images for: Electrodiffusion dynamics in the cardiomyocyte dyad at nano-scale resolution using the Poisson-Nernst-Planck (PNP) equations
Source: PLoS Comput Biol. 2025 Jun 12;21(6):e1013149. doi: 10.1371/journal.pcbi.1013149 (PMC12187020; doi:10.1371/journal.pcbi.1013149)

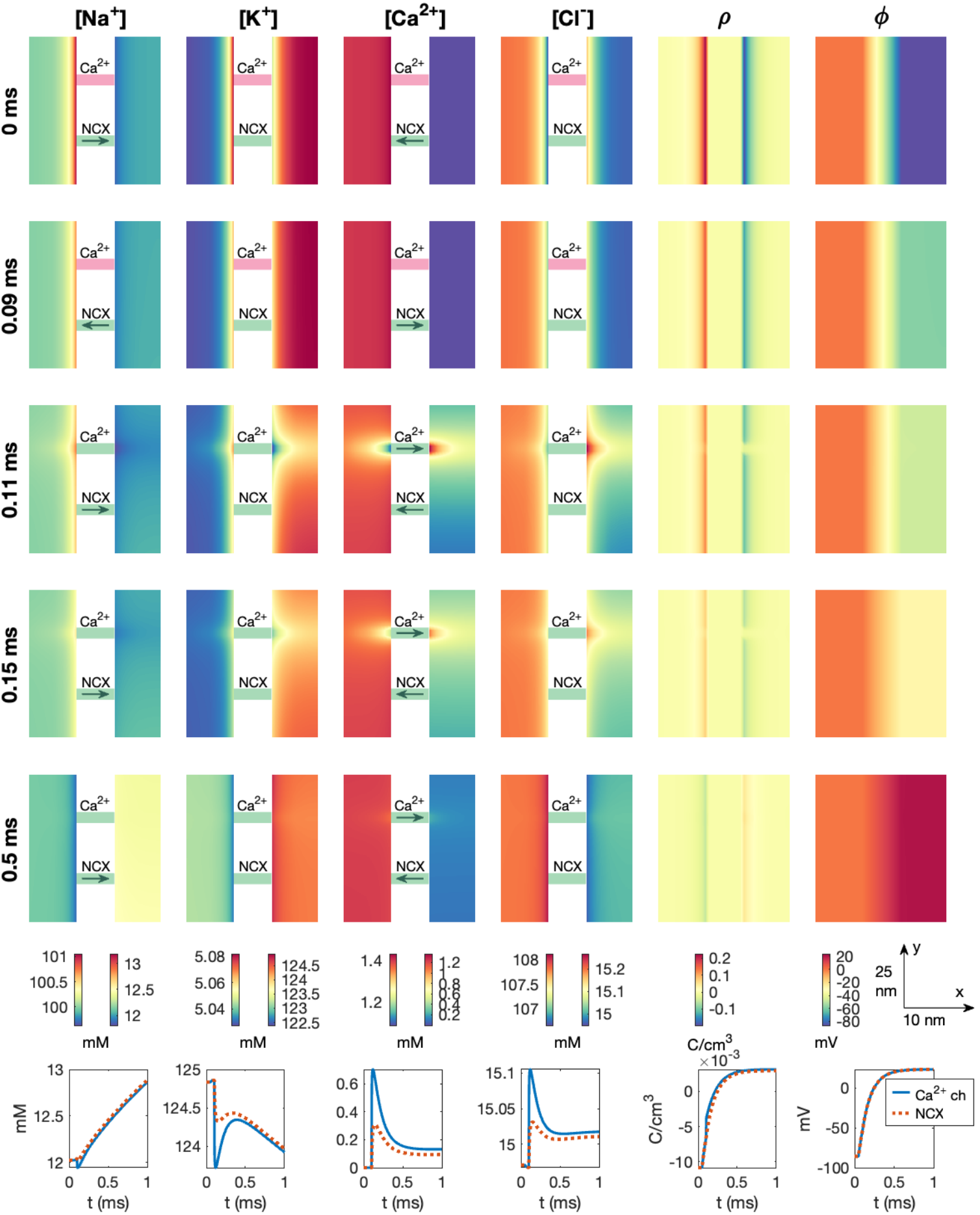

Supplement: S1 Fig — The simulation is the same as that displayed in Fig 12 in the main paper, except that there are no Ca2+ binding buffers present. (PDF) [file pcbi.1013149.s001.pdf]

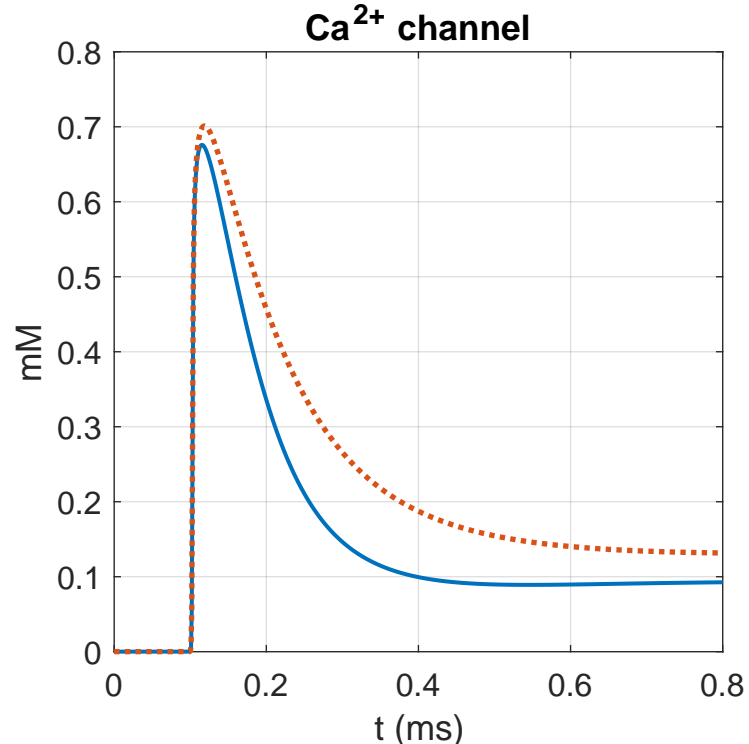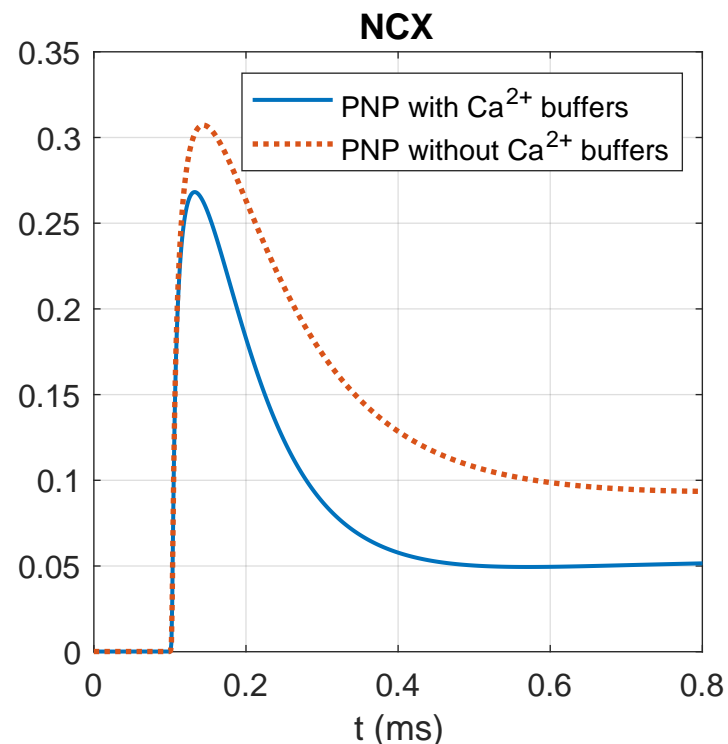

Supplement: S2 Fig — The simulation with Ca2+ buffers is displayed in Fig 12 in the main paper and the simulation without Ca2+ buffers is displayed in S1 Fig. (PDF) [file pcbi.1013149.s002.pdf]
