# Supplementary material for: Electrodiffusion dynamics in the cardiomyocyte dyad at nano-scale resolution using the Poisson-Nernst-Planck (PNP) equations
Source: PLoS Comput Biol. 2025 Jun 12;21(6):e1013149. doi: 10.1371/journal.pcbi.1013149 (PMC12187020; doi:10.1371/journal.pcbi.1013149)
Supplement: S1 Appendix — (PDF) [file pcbi.1013149.s003.pdf]

S1 Appendix: Representing ion channels as membrane pores

Figures I and II compares the results of simulations of an open K^+ channel when the channel is represented as selectively open for diffusion (Figure I) and when the channel is represented by internal boundary conditions (Figure II). Note that Figure II displays the same results as Figure 7 in the main paper, but in Figure II the scaling of the colormaps are adjusted to be the same as for Figure I.

When the channel is represented as selectively open for diffusion (Figure I), the concentration of all the ionic species except for the one that is able to move through the channel is set to zero. Furthermore, the background charge density, ρ_0, is set up such that ρ is initially zero everywhere (including in the channel). Thus, ρ_0 in the channel is set up to directly counter the initial condition set up in the channel. In the simulation displayed in Figure I, the initial concentration of K^+ in the K^+ channel is set up as a linear function of x between the intracellular and extracellular concentrations. But, as observed in [1], other initial concentration profiles could have been selected, changing the dynamics close to the channel somewhat. Furthermore, in the simulation displayed in Figure I, the diffusion coefficient for K^+ was set to $d_{K^+} = 1.66 \cdot 10^4$ nm^2/ms in the K^+ channel. This value was selected because it made the duration of the dynamics similar to the simulation in Figure II, where the K^+ channel conductance was set to a physiologically realistic value of $g_{K^+} = 5$ pS.

Comparing Figures I and II, we observe that the dynamics appear to be quite similar for the two different channel representations. For example, the intracellular potential approaches a value of about -80 mV in what appears to be the same timespan. Moreover, the K^+ concentration changes locally near the channel and the other ionic species are also changed in these locations to counteract the deviation from electroneutrality. Furthermore, at rest, a Debye layer is present in both cases. On the other hand, a few differences between the two cases are also evident. For instance, the time evolution of the intracellular K^+ concentration 3.5 nm outside of the K^+ channel (lower panel) is a bit different in the two cases. The charge density, ρ, and the potential, ϕ, in the K^+ channel is also different between the two cases.

References

[1] Karoline Horgmo Jæger, Ena Ivanovic, Jan P Kucera, and Aslak Tveito. Nano-scale solution of the Poisson-Nernst-Planck (PNP) equations in a fraction of two neighboring cells reveals the magnitude of intercellular electrochemical waves. *PLoS Computational Biology*, 19(2):e1010895, 2023.

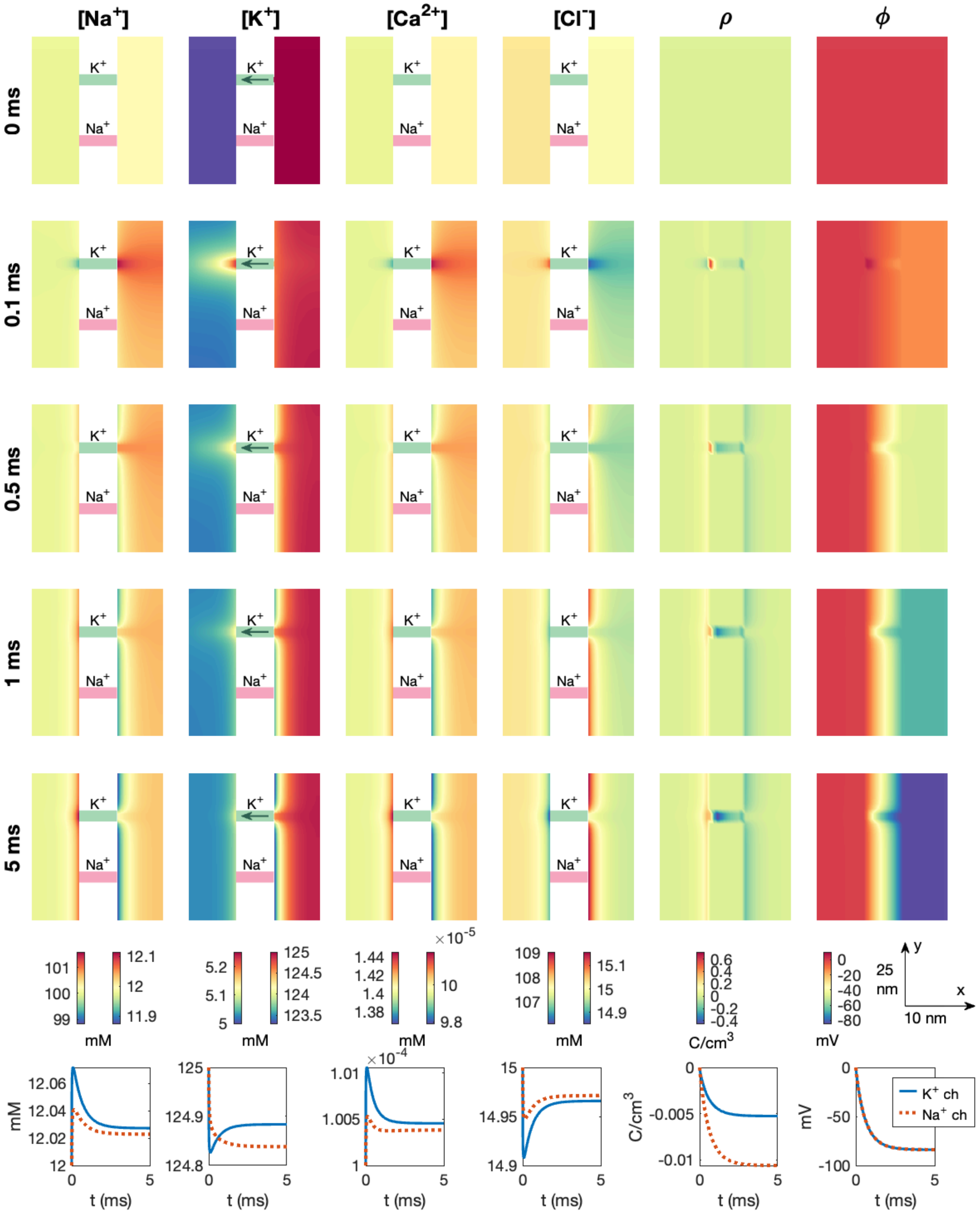

Figure I: **Dynamics following the opening of a K^+ channel in a PNP model simulation with the channel represented like a membrane pore.** We use $\Delta t = 1$ μs and an adaptive mesh.

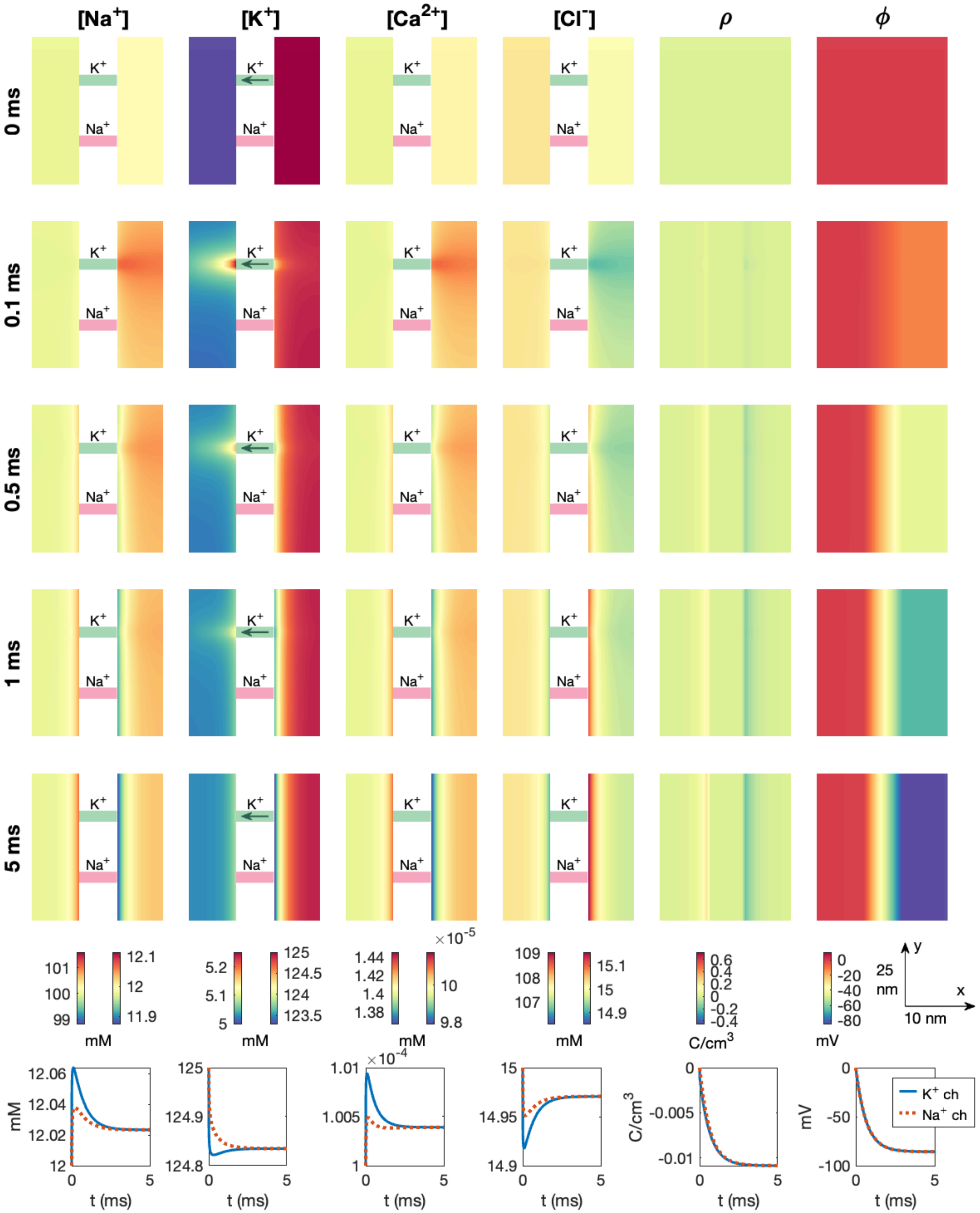

Figure II: **Dynamics following the opening of a K^+ channel in a PNP model simulation with the channel represented using internal boundary conditions** This is the same simulation as in Figure 7, but the scaling of the colorbars are adjusted such that they match the ones used in Figure I. We use $\Delta t = 1$ μs and an adaptive mesh.
