# Supplementary material for: Electrodiffusion dynamics in the cardiomyocyte dyad at nano-scale resolution using the Poisson-Nernst-Planck (PNP) equations
Source: PLoS Comput Biol. 2025 Jun 12;21(6):e1013149. doi: 10.1371/journal.pcbi.1013149 (PMC12187020; doi:10.1371/journal.pcbi.1013149)
Supplement: S2 Appendix — (PDF) [file pcbi.1013149.s004.pdf]

S2 Appendix: Convergence of the numerical scheme for the PNP model

In Figure I, we investigate the convergence of the numerical scheme applied in our simulations. We consider the example case depicted in Figure 4 of the main paper. In the left panel of Figure I, we consider the solutions for different values of Δt and in the right panel, we consider the solutions for different values of $\Delta x = \Delta y$. We observe that the solutions converge as the time step is reduced.

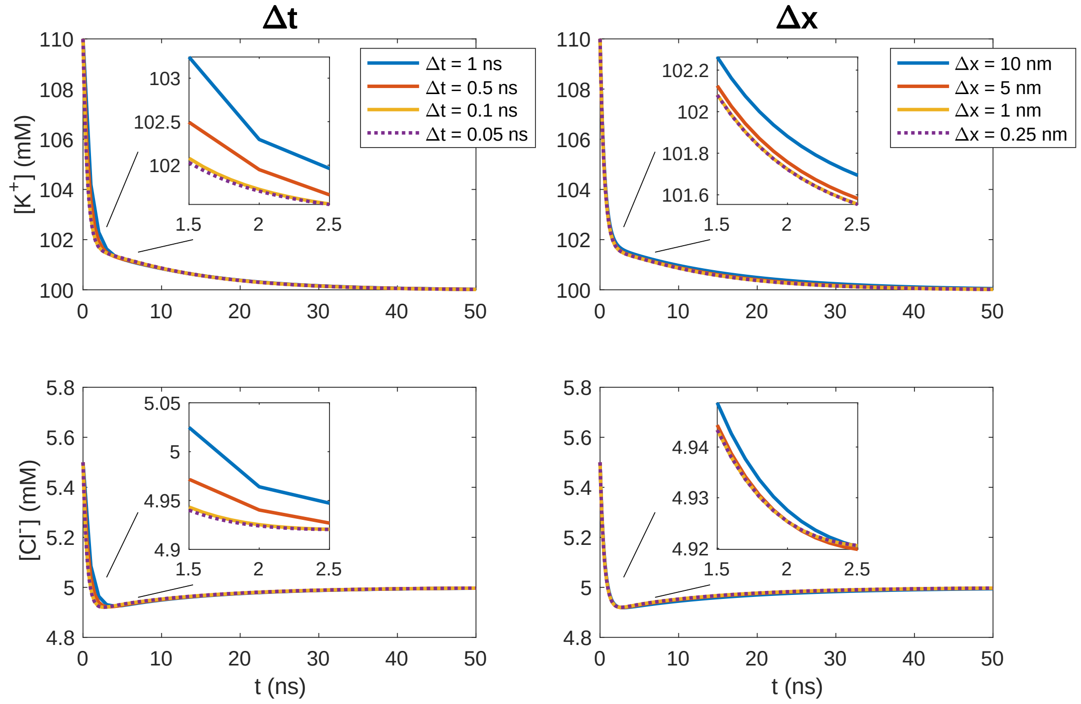

Figure I: **Convergence of the numerical scheme.** We consider the example case depicted in Figure 4 in the main paper and plot the ionic concentrations in the center point of the domain. In the left panel, we consider the solutions for different values of Δt and keep $\Delta x = \Delta y$ fixed at the default value of 0.25 nm, and in the right panel, we consider the solutions for different values of $\Delta x = \Delta y$ and keep Δt fixed at 0.1 ns.

For the dyad simulations involving open channels considered later in the paper, the perturbations to the ionic concentrations are more gradual than the large initial perturbations considered here. Consequently, these simulations allow for larger time steps than the ones used in Figure I. In Figure II, we consider a 3D simulation where the K^+, Na^+ and Ca^{2+} channels and the NCX are all open (equivalent to Figure 12 in the main paper) for some different values of Δt. We observe that the solutions appears to be relatively converged at time steps of about $\Delta t = 1$ μs.

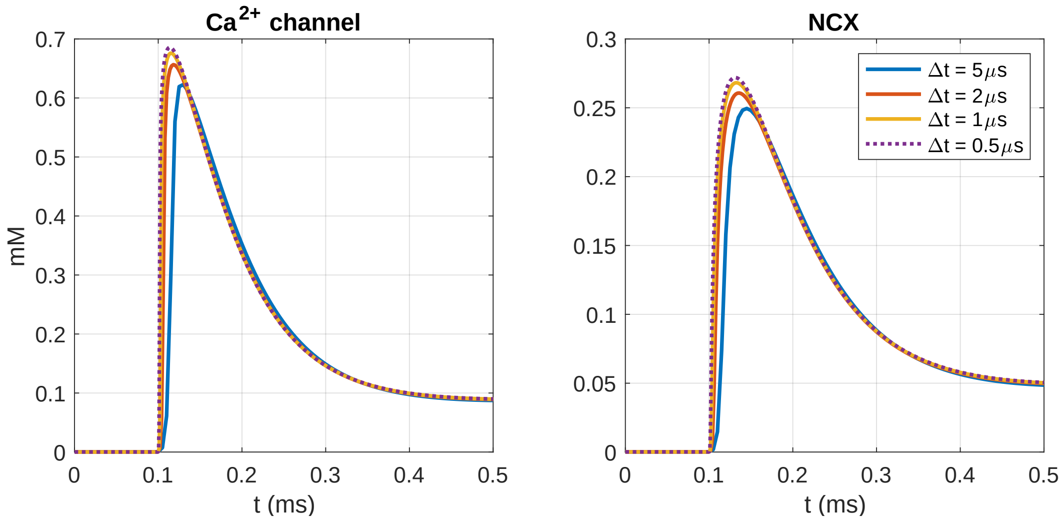

Figure II: **Ca^{2+} concentration 3.5 nm to the left of the Ca^{2+} channel and the NCX for different values of Δt.** We consider the simulation displayed in Figure 12 in the main paper for different values of Δt.
