# Supplementary material for: Electrodiffusion dynamics in the cardiomyocyte dyad at nano-scale resolution using the Poisson-Nernst-Planck (PNP) equations
Source: PLoS Comput Biol. 2025 Jun 12;21(6):e1013149. doi: 10.1371/journal.pcbi.1013149 (PMC12187020; doi:10.1371/journal.pcbi.1013149)
Supplement: S3 Appendix — (PDF) [file pcbi.1013149.s005.pdf]

S3 Appendix: Comparison of equation term sizes for Na^+

In Figure 18 in the main paper, we observed that the diffusion term, B_d, was much larger than the electrical term, B_e, in the equation governing the Ca^{2+} concentration in the dyad following the opening of a membrane Ca^{2+} channel. As a result, the Ca^{2+} concentration solution of the PNP model and the pure reaction-diffusion model was virtually identical (see Figure 17 in the main paper and the left panel of Figure II).

In Figure I, we similarly plot the terms in the equation governing the Na^+ concentration following the opening of a membrane Na^+ channel. Note that we have not included Na^+ binding buffers in our model, so the term B_b is zero for Na^+. We observe that for Na^+, the size of the electrical term, B_e, is more similar to the size of the diffusion term, B_d. In the right panel of Figure II, we observe that this results in a larger difference between the solution of the PNP model and the pure reaction-diffusion model.

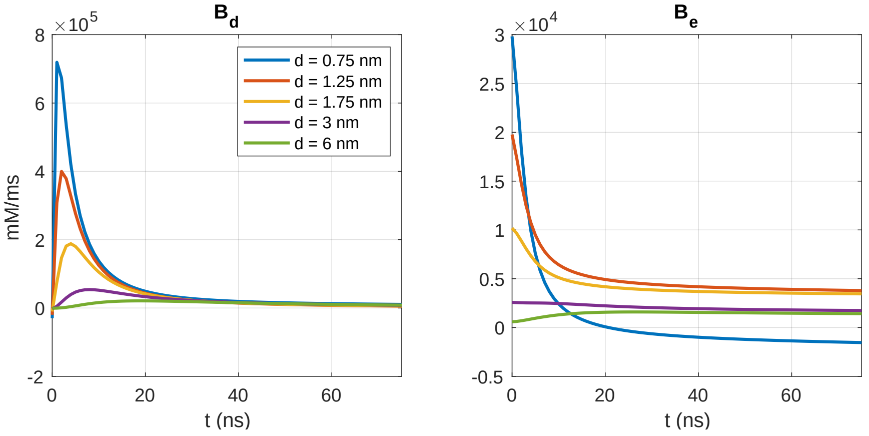

Figure I: **Terms in the PNP model equation for the Na^+ concentration.** We consider the solution in the first 80 ns of simulation after the Na^+ channel is opened in points at different distances, d, in the x-direction from the intracellular mouth of the Na^+ channel. We have used the default model parameters, $\Delta t = 1$ ns, and an adaptive mesh.

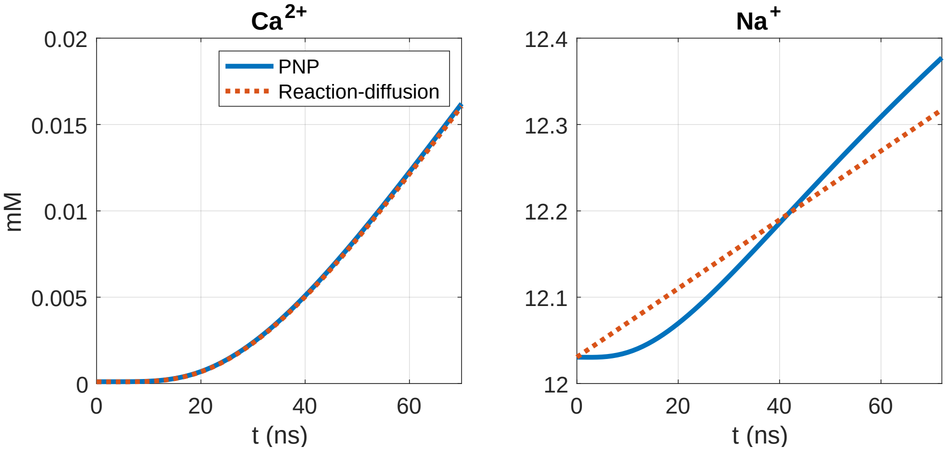

Figure II: **Concentration solutions for the PNP and pure reaction-diffusion models.** In the left panel, we consider the Ca^{2+} concentration following the opening of a Ca^{2+} channel, and in the right panel, we consider the Na^{+} concentration following the opening of an Na^{+} channel. We consider the average concentrations in an area spanning 30 nm $\times$ 30 nm on the SR membrane directly across from the opened channel.
