# Supplementary material for: Electrodiffusion dynamics in the cardiomyocyte dyad at nano-scale resolution using the Poisson-Nernst-Planck (PNP) equations
Source: PLoS Comput Biol. 2025 Jun 12;21(6):e1013149. doi: 10.1371/journal.pcbi.1013149 (PMC12187020; doi:10.1371/journal.pcbi.1013149)
Supplement: S4 Appendix — (PDF) [file pcbi.1013149.s006.pdf]

S4 Appendix: ODE model representations

In this supplementary note, the ODE model representations of the Ca^{2+} dynamics in the dyad that are compared to the PNP model are described.

1 ODE model with one dyad compartment

The ODE model for one dyad compartment reads

$$\frac{dc_d}{dt} = \frac{1}{V_d}\left(J_{\text{ch}} - J_{d,l}\right) - \sum_{j\in B_d} J_{B_{d,j}}, \tag{1}$$

$$\frac{dc_l}{dt} = \frac{1}{V_l} J_{d,l} - \sum_{j\in B_l} J_{B_{l,j}}, \tag{2}$$

$$\frac{db_{d,j}}{dt} = J_{B_{d,j}}, \tag{3}$$

$$\frac{db_{l,j}}{dt} = J_{B_{l,j}}, \tag{4}$$

$$J_{B_{d,j}} = k_{\text{on}}^{d,j} c_d \left(B_{\text{tot}}^{d,j} - b_{d,j}\right) - k_{\text{off}}^{d,j} b_{d,j}, \tag{5}$$

$$J_{B_{l,j}} = k_{\text{on}}^{l,j} c_l \left(B_{\text{tot}}^{l,j} - b_{l,j}\right) - k_{\text{off}}^{l,j} b_{l,j}. \tag{6}$$

Here, c_d is the Ca^{2+} concentration and $b_{d,j}$ are the concentrations of Ca^{2+} bound to a buffer in the dyad. Likewise, c_l is the Ca^{2+} concentration and $b_{l,j}$ are the concentrations of Ca^{2+} bound to a buffer in the large cytosolic compartment. The concentrations have unit mM. The parameters V_d and V_l represent the volumes of the two compartments (in nm^3), and B_d and B_l are collections of Ca^{2+} binding buffers in Ω_d and Ω_l, respectively. To make the comparison to the PNP model more straightforward, we consider the same buffers as in the PNP model in both compartments.

The Ca^{2+} channel flux, J_{ch} (in mMnm^3/ms), is defined as

$$J_{\text{ch}} = \frac{1}{2F} g_{\text{Ca}^{2+}}(v - v_{0,\text{Ca}^{2+}}). \tag{7}$$

Here, $v_{0,\text{Ca}^{2+}}$ is the Nernst equilibrium potential of Ca^{2+} defined in the main paper. In the ODE model simulations, the intracellular concentration is defined as c_d and the extracellular concentration is fixed at the value provided for the initial condition for extracellular Ca^{2+} concentration. Furthermore, v is taken from the corresponding PNP model simulation.

The diffusion flux, $J_{d,l}$ (in mMnm^3/ms), is defined as

$$J_{d,l} = \frac{D_{\text{Ca}^{2+}} A_{d,l}}{L_{d,l}}(c_d - c_l), \tag{8}$$

where $D_{\text{Ca}^{2+}}$ is the intracellular diffusion coefficient for Ca^{2+}, $A_{d,l}$ (in nm^2) is the average cross-sectional area connecting compartments d and l, and $L_{d,l}$ (in nm) is the distance between the centers of the two compartments.

Parameter	Value
L_d (one dyad comp.)	200 nm
L_d (two dyad comp.)	90 nm
$L_{d,l}$, $L_{c,l}$, $L_{r,l}$	400 nm
V_l	$1 \cdot 10^7$ nm^3

Table I: **Parameter values used for the ODE model.** The remaining parameter values are found in the tables of the main paper text. The value of L_d is fitted to match the RyR activation time of the PNP model in the case $D_{\text{Ca}^{2+}} = 200\ 000$ nm^2/ms and $L_i = 7$ nm. The remaining parameter values are rough estimates.

The parameter values of the ODE model are given in Table **??**. Note that the dyad is assumed to be a volume of size

$$V_d = L_i L_d^2, \tag{9}$$

where L_i is the dyad width, and L_d defines the size of the dyad in the two other spatial directions. The value of L_d is fitted such that the time from Ca^{2+} channel opening to RyR threshold is roughly the same as for the PNP model for $D_{\text{Ca}^{2+}} = 200\ 000$ nm^2/ms and $L_i = 7$ nm. Furthermore, $A_{d,l}$ is defined as the intersection area between the dyad and the large compartment, i.e.,

$$A_{d,l} = 4 L_i L_d. \tag{10}$$

2 ODE model with two dyad compartments

The ODE model for two dyad compartments reads

$$\frac{dc_c}{dt} = \frac{1}{V_c}\left(J_{\text{ch}} - J_{c,l} - J_{c,r}\right) - \sum_{j \in B_c} J_{B_{c,j}}, \tag{11}$$

$$\frac{dc_r}{dt} = \frac{1}{V_r}\left(J_{c,r} - J_{r,l}\right) - \sum_{j \in B_r} J_{B_{r,j}}, \tag{12}$$

$$\frac{dc_l}{dt} = \frac{1}{V_l}(J_{c,l} + J_{r,l}) - \sum_{j \in B_l} J_{B_{l,j}}, \tag{13}$$

$$\frac{db_{c,j}}{dt} = J_{B_{c,j}}, \tag{14}$$

$$\frac{db_{r,j}}{dt} = J_{B_{r,j}}, \tag{15}$$

$$\frac{db_{l,j}}{dt} = J_{B_{l,j}}, \tag{16}$$

$$J_{B_{c,j}} = k_{\text{on}}^{c,j} c_c \left(B_{\text{tot}}^{c,j} - c_{c,j}\right) - k_{\text{off}}^{c,j} b_{c,j}, \tag{17}$$

$$J_{B_{r,j}} = k_{\text{on}}^{r,j} c_r \left(B_{\text{tot}}^{r,j} - b_{r,j}\right) - k_{\text{off}}^{r,j} b_{r,j}, \tag{18}$$

$$J_{B_{l,j}} = k_{\text{on}}^{l,j} c_l \left(B_{\text{tot}}^{l,j} - b_{l,j}\right) - k_{\text{off}}^{l,j} b_{l,j}. \tag{19}$$

Here, c_c is the dyad Ca^{2+} concentration in the compartment close to the membrane Ca^{2+} channel, c_r is the dyad Ca^{2+} concentration in the compartment close to the RyR, and c_l is the Ca^{2+} concentration in the surrounding large cytosol compartment. The units and definitions of the remaining variables and parameters follow the same convention as for the ODE model with one dyad compartment.

The diffusion fluxes are defined as

$$J_{c,r} = \frac{D_{\mathrm{Ca}^{2+}} A_{c,r}}{L_{c,r}} (c_c - c_r), \tag{20}$$

$$J_{c,l} = \frac{D_{\mathrm{Ca}^{2+}} A_{c,l}}{L_{c,l}} (c_c - c_l), \tag{21}$$

$$J_{r,l} = \frac{D_{\mathrm{Ca}^{2+}} A_{r,l}}{L_{r,l}} (c_r - c_l). \tag{22}$$

Moreover, we use the following assumptions regarding the compartment geometries,

$$A_{c,r} = L_d^2, \qquad A_{c,l} = A_{r,l} = \frac{A_{d,l}}{2} = 2L_i L_d, \tag{23}$$

$$L_{c,r} = \frac{L_i}{2}, \qquad V_c = V_r = \frac{V_d}{2} = \frac{L_i L_d^2}{2}. \tag{24}$$
