# Supplementary material for: Electrodiffusion dynamics in the cardiomyocyte dyad at nano-scale resolution using the Poisson-Nernst-Planck (PNP) equations
Source: PLoS Comput Biol. 2025 Jun 12;21(6):e1013149. doi: 10.1371/journal.pcbi.1013149 (PMC12187020; doi:10.1371/journal.pcbi.1013149)
Supplement: S5 Appendix — (PDF) [file pcbi.1013149.s007.pdf]

S5 Appendix: Dyad dynamics in a PNP model incorporating steric effects

In this supplementary note, we investigate how the dynamics are affected when the dyad becomes crowded. This is done by solving the PNP equations with steric effects included.

1 The PNP model with steric effects

We apply the PNP model including steric effects from [1]. The model for the ion concentrations with steric effects from [1] takes the form

$$\frac{\partial p_k}{\partial t} = \nabla \cdot (D_k \nabla p_k) + \nabla \cdot \left(\frac{D_k z_k e}{k_B T} p_k \nabla \phi \right) + \nabla \cdot \left(\frac{D_k k_k p_k \sum_l a_l^3 \nabla p_l}{1 - \sum_l a_l^3 p_l} \right). \tag{1}$$

Here, p_k is the density of a type of ion (in 1/L), a_k is the length of the ion type (in dm) and $k_k = (a_k/a_0)^3$, where a_0 is the solvent molecule size (in dm).

To rewrite this equation to a form more similar to what we have used in this study, we note that the ion density p_k (in 1/L) can be converted to the ion concentration (in mM) by division by Avogadro's constant: $N_A = 6.02214076 \cdot 10^{20}$ mmol^{-1}. More specifically,

$$p_k = N_A c_k. \tag{2}$$

Inserting this in (1) and dividing both sides of the equation by N_A, we obtain

$$\frac{\partial c_k}{\partial t} = \nabla \cdot (D_k \nabla c_k) + \nabla \cdot \left(\frac{D_k z_k e}{k_B T} c_k \nabla \phi \right) + \nabla \cdot \left(\frac{D_k k_k c_k \sum_l a_l^3 N_A \nabla c_l}{1 - \sum_l a_l^3 N_A c_l} \right). \tag{3}$$

To simplify, we now define

$$\lambda_k = N_A a_k^3. \tag{4}$$

The unit of λ_k is mmol^{-1}dm^3=(mM)$^{-1}$. Inserting (4) in (3), we get

$$\frac{\partial c_k}{\partial t} = \nabla \cdot (D_k \nabla c_k) + \nabla \cdot \left(\frac{D_k z_k e}{k_B T} c_k \nabla \phi \right) + \nabla \cdot \left(\frac{D_k k_k c_k \sum_l \lambda_l \nabla c_l}{1 - \sum_l \lambda_l c_l} \right). \tag{5}$$

2 Including buffers

In the case when ion binding buffering proteins are included, the model includes the ionic concentration, c_k, the concentration of ions bound to the buffer, $b_{k,j}$ and the concentration of buffer with no ion bound, $B_{\text{tot}}^{k,j}$. Assuming that a_k is the size of the ion and a_B is the size of the buffer protein (with no ion bound), we can define λ_k and λ_B like in (4). If we have only one ion species, k, and one buffering protein, j, the sum in the denominator of the last term in (5) reads

$$\begin{aligned}\sum_l \lambda_l c_l &= \lambda_k c_k + (\lambda_k + \lambda_B) b_{k,j} + \lambda_B (B_{\text{tot}}^{k,j} - b_{k,j}) \\ &= \lambda_k (c_k + b_{k,j}) + \lambda_B B_{\text{tot}}^{k,j}.\end{aligned}$$

Parameter	Value	Reference
$a_{\mathrm{Ca}^{2+}}$	0.39 nm $= 3.9 \cdot 10^{-9}$ dm	[2]
$a_{\mathrm{Na}^{+}}$	0.38 nm $= 3.8 \cdot 10^{-9}$ dm	[2]
$a_{\mathrm{K}^{+}}$	0.49 nm $= 4.9 \cdot 10^{-9}$ dm	[2]
$a_{\mathrm{Cl}^{-}}$	0.16 nm $= 1.6 \cdot 10^{-9}$ dm	[2]
a_0	0.3 nm $= 3 \cdot 10^{-9}$ dm	[3]
a_B	5 nm $= 5 \cdot 10^{-8}$ dm	[4]

Table I: **Parameter values used for the PNP model including steric effects.** Note that a_0 is the size of the solvent molecule, i.e., water, and a_B is the size of a Ca^{2+} buffer protein. The remaining parameter values are found in the tables of the main paper.

Since $B_{\mathrm{tot}}^{k,j}$ is constant both in time and space in the intracellular domain, the sum in the nominator reads

$$\begin{aligned}\sum_l \lambda_l \nabla c_l &= \lambda_k \nabla c_k + (\lambda_k + \lambda_B)\nabla b_{k,j} + \lambda_B \nabla (B_{\mathrm{tot}}^{k,j} - b_{k,j}) \\ &= \lambda_k (\nabla c_k + \nabla b_{k,j}).\end{aligned}$$

More generally, including buffers, the last term of (5) reads

$$\nabla \cdot \left(\frac{D_k k_k c_k \sum_l \lambda_l \left(\nabla c_l + \sum_{j \in B_l} \nabla b_{l,j} \right)}{1 - \sum_l \left(\lambda_l \left(c_l + \sum_{j \in B_l} b_{l,j} \right) + \sum_{j \in B_l} \lambda_{B_{l,j}} B_{\mathrm{tot}}^{l,j} \right)} \right).$$

3 Full system of equations

The full system of equations for the PNP model with steric effects and buffers reads

$$\nabla \cdot (\varepsilon_r \varepsilon_0 \nabla \phi) = -\rho, \tag{6}$$

$$\begin{aligned}\frac{\partial c_k}{\partial t} &= \nabla \cdot D_k \nabla c_k + \nabla \cdot \left(\frac{D_k z_k e}{k_B T} c_k \nabla \phi \right) - \sum_{j \in B_k} J_{B_{k,j}} \\ &+ \nabla \cdot \left(\frac{D_k k_k c_k \sum_l \lambda_l \left(\nabla c_l + \sum_{j \in B_l} \nabla b_{l,j} \right)}{1 - \sum_l \left(\lambda_l \left(c_l + \sum_{j \in B_l} b_{l,j} \right) + \sum_{j \in B_l} \lambda_{B_{l,j}} B_{\mathrm{tot}}^{l,j} \right)} \right),\end{aligned} \tag{7}$$

$$\frac{\partial b_{k,j}}{\partial t} = J_{B_{k,j}}, \tag{8}$$

$$\rho = \rho_0 + F \sum_k z_k \left(c_k + \sum_j b_{k,j} \right), \tag{9}$$

where the last term of (9) represents the steric effects. Note here that

$$\lambda_k = N_A a_k^3, \tag{10}$$

$$\lambda_{B_{l,j}} = N_A a_B^3, \tag{11}$$

$$k_k = (a_k / a_0)^3. \tag{12}$$

The parameter values applied for the parameters a_k and a_B are given in Table I.

4 Calcium dynamics in the dyad for the PNP model including steric effects

In Figure I we have performed a simulation similar to the one displayed in Figure 12 using the PNP model with steric effects included. We observe that including steric effects does not seem to have a significant effects on the computed dynamics (compare to Figure 12 in the main paper). Indeed, in Figure II, we compare the Ca^{2+} concentration in points 3.5 nm to the left of the Ca^{2+} channel and the NCX for the PNP model with and without steric effects, and no visible difference is present.

References

[1] Benzhuo Lu and YC Zhou. Poisson-Nernst-Planck equations for simulating biomolecular diffusion-reaction processes II: Size effects on ionic distributions and diffusion-reaction rates. *Biophysical Journal*, 100(10):2475–2485, 2011.

[2] LibreTexts. Chemistry — the central science. `https://chem.libretexts.org/Bookshelves/General_Chemistry/Map%3A_Chemistry_-_The_Central_Science_(Brown_et_al.)/07%3A_Periodic_Properties_of_the_Elements/7.03%3A_Sizes_of_Atoms_and_Ions`, 2025.

[3] Malin Edvardsson. What is nanotechnology? `https://www.biolinscientific.com/blog/what-is-nanotechnology`, 2020.

[4] Antti J Tanskanen, Joseph L Greenstein, Alex Chen, Sean X Sun, and Raimond L Winslow. Protein geometry and placement in the cardiac dyad influence macroscopic properties of calcium-induced calcium release. *Biophysical Journal*, 92(10):3379–3396, 2007.

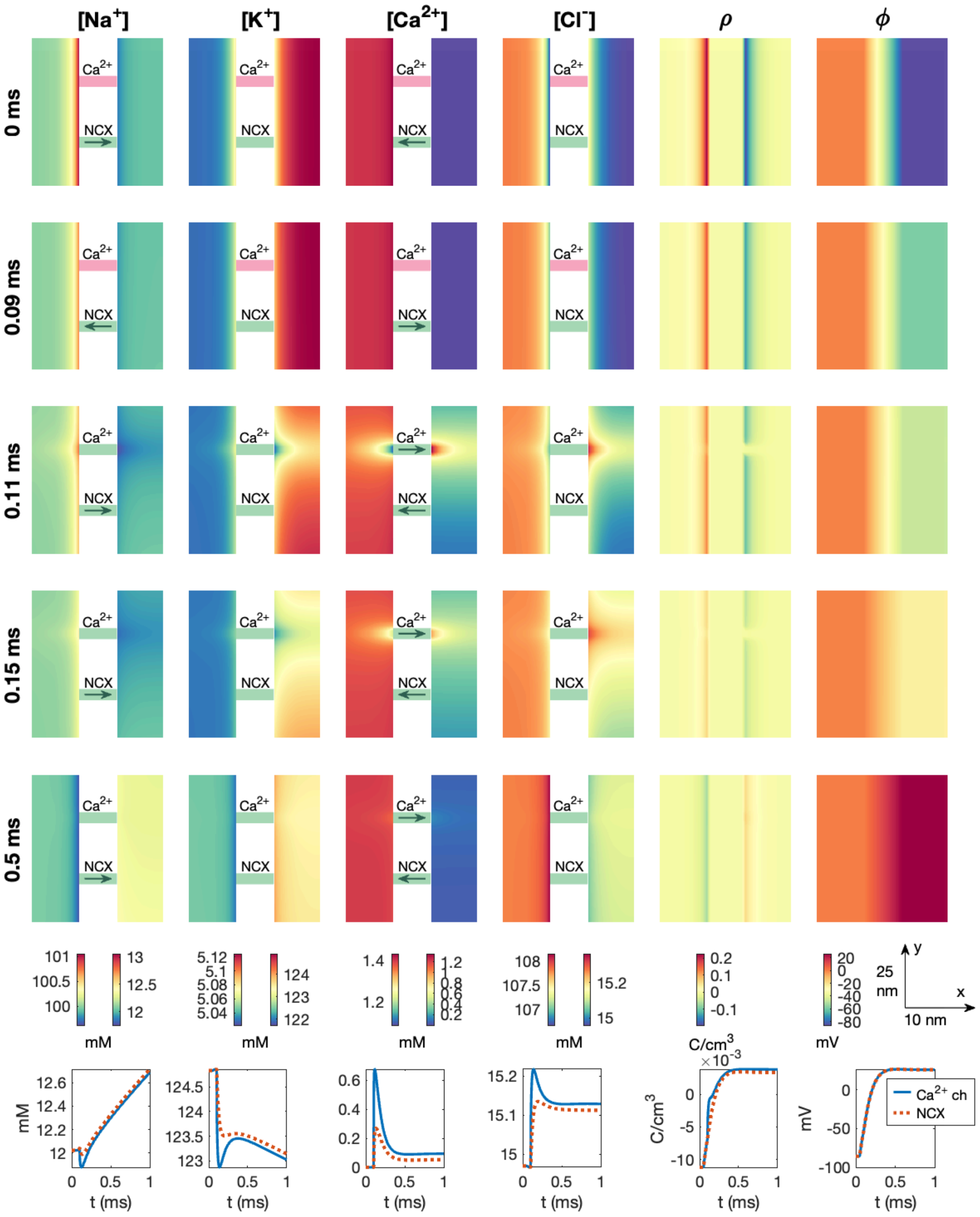

Figure I: **Dynamics following the opening of a Ca^{2+} channel in a PNP model simulation with steric effects.** The simulation is the same as that displayed in Figure 12 in the main paper, except that steric effects are included in the model as described in (6)–(8).

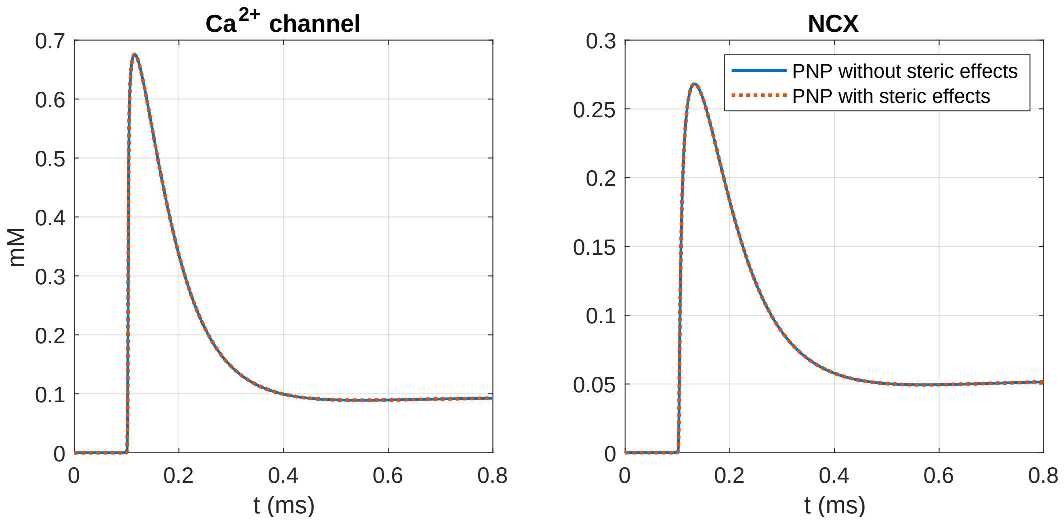

Figure II: **Ca^{2+} concentration 3.5 nm to the left of the Ca^{2+} channel and the NCX.** We consider the simulation displayed in Figure 12 in the main paper and the simulation displayed in Figure I.
