# Supplementary material for: Electrodiffusion dynamics in the cardiomyocyte dyad at nano-scale resolution using the Poisson-Nernst-Planck (PNP) equations
Source: PLoS Comput Biol. 2025 Jun 12;21(6):e1013149. doi: 10.1371/journal.pcbi.1013149 (PMC12187020; doi:10.1371/journal.pcbi.1013149)
Supplement: Attachment — Submitted filename: review_PCOMPBIOL-D-25-00509.pdf [file pcbi.1013149.s008.pdf]

1 Review of “Electrodiffusion in the dyad at nano-scale resolution using the PNP equations” by K. H. Jæger and A. Tveito.

This manuscript presents an implementation of an electro-diffusion model based on the Poisson-Nernst-Planck equations in the dyadic space of cardiomyocites, where the SR organelle, source of calcium ions, is in close proximity to the cell membrane. In fact the specific implementation is adapted from an earlier study by the same authors [1], where the electro-chemical coupling of two cardiomyocites was considered. Here instead of considering two distinct intracellular spaces and membranes separated by a thin extracellular space, the domain consists of single intra- and extracellular spaces connected via a thin membrane with embedded ionic channels. I think the manuscript will be of interest for the readership of PLOS Computational Biology and thus recommend its publication. It clearly fits within the journal scope, plus it is generally well-written and easy to follow (with detailed descriptions of boundary conditions and of how to deal with ionic channels). It will be accessible to a wide audience of computational biologists and numerical analysts looking for applications. However several aspects need to be clarified and polished: main message, figures, physiological applications, etc. In its current form, the paper looks like a collection of numerical experiments lacking a clear conducting thread. I hope the suggestions below well help the authors improving the manuscript and its message.

- **Dyadic space.** It is unclear how the electro-diffusion model presented here specifically applies to the dyadic space of cardiomyocites. Fig. 8 suggests that the rightward boundary of the intracellular domain would correspond to the sarcoplasmic reticulum membrane, but my understanding is that SR feedback and influence are not considered. As it is, the model could well describe generic ionic electro-diffusion dynamics near membrane-bound channels of subcellular domains of all types.
  - I see that numerical experiments specific to the cardiac dyad are presented in section 3.9 (ryanodine receptors activation time, dyad width, . . .), but these should be presented early on I think.
- **Membrane curvature.** Can the numerical methodology presented here handle curved boundaries? Would that be relevant for dyadic space, where the SR structure presents highly irregular shape and morphologies?
- **Figures.** Would it be possible to provide more concise and synthetic figures, that would be more attractive to the reader? It’s fine to have a short accompanying caption with the figure, but where is the need to add lengthy descriptions of each subplot and subfigure as in Fig. 4 (**A:**, **B:**, **C:**, . . .) ? I feel many figures also contain redundant information, and the authors could focus on one ionic species density, with only one solution snapshot.
- **Buffers and permanent charges.** One difference with the earlier model [1] is the inclusion of calcium buffers. Although their role and importance are clear from a modeling point of view, can the authors comment (and quantify) the influence of buffers on PNP numerical solutions? What differences do we see with and without buffers? Same question for the permanent charge density ρ from Eq. (8).
- **Diffusion model.** Some conclusions about the pure diffusion model formation seems obvious to me. . . Of course by neglecting the electric field (section 3.10), no Debye layer near the membrane can be formed. It becomes a bit more interesting at the end of page 28, where the authors provide a reference on how to account for the voltage in a pure diffusion model. What is again the purpose of this section (3.10)? This remains unclear to me.

References

[1] K. H. Jaeger, E. Ivanovic, J. P. Kucera, and A. Tveito, “Nano-scale solution of the poisson-nernst-planck (pnp) equations in a fraction of two neighboring cells reveals the magnitude of intercellular electrochemical waves,” *PLOS Computational Biology*, vol. 19, pp. 1–31, 02 2023.
