# Supplementary material for: Electrodiffusion dynamics in the cardiomyocyte dyad at nano-scale resolution using the Poisson-Nernst-Planck (PNP) equations
Source: PLoS Comput Biol. 2025 Jun 12;21(6):e1013149. doi: 10.1371/journal.pcbi.1013149 (PMC12187020; doi:10.1371/journal.pcbi.1013149)
Supplement: Attachment — Submitted filename: Response.pdf [file pcbi.1013149.s009.pdf]

Response to reviewer comments

The authors deeply appreciate the efforts of the reviewers. We are impressed and grateful for the thorough reviews, and we have aimed to carefully address every point raised.

Reviewer #1:

In the present paper, the authors study electrodiffusion dynamics near the cell membrane of a cardiac cell. To model the electrical properties of cardiac cells, normally either average concentrations, or spatial concentrations following reaction-diffusion equations have been considered in the literature. A pertinent question, that the authors try to answer in this paper (and that I have also asked myself) is whether electrical effects are important at short spatial and/or temporal scales. To study these effects, the authors use Poisson-Nernst-Planck (PNP) equations, and compare their results with those obtained using a pure reaction-diffusion scheme, or a model of compartments. Quite reassuring, they find that, although a Debye layer is formed near the surface, the reaction-diffusion equations are able to describe correct many phenomena of interest.

As I said, I find this a very relevant paper to understand the limits of the models normally used for describing the electrical properties of cardiac cells. This study has also its limits (use of a continuous description, stochastic dynamics of the ion channels, phenomenological buffers, etc). The authors are well aware of these limitations and discuss them in the final section of the paper. Maybe another aspect that the authors could include in the discussion is the number of channels in the model. If I understand correctly, the authors consider an area of the cell membrane of 1 microm^2. In this area there should be a decent number of channels. In fact, some channels come often in clusters. The authors should discuss this point and whether it could affect their results.

Authors' response:

Thank you for these positive remarks. We agree that 1um^2 is sufficient to accommodate multiple ion channels of each type, and that, physiologically, channels often occur in clusters. However, in our model setup, the number of channels was adjusted to the area under consideration to achieve approximately correct upstroke velocity. For the chosen membrane area of 1um^2 (1000nm x 1000nm) we found that including a single sodium channel yields an upstroke velocity in line with physiological expectations. This choice reflects the trade-off between biological realism and numerical tractability in the present study.

We have added the following sentences to the *Discussion* section (Section 4.5):

Also, ion channels in physiological membranes often appear in clusters rather than as isolated entities, see, e.g., [53, 54], but for simplicity, we have used only one channel of each type in our simulations. This entails one Na^+ channel per 1 μm^2, tuned to reproduce a realistic upstroke velocity within the modeled domain.

Reviewer #2:

This manuscript presents an implementation of an electro-diffusion model based on the Poisson-Nernst- Planck equations in the dyadic space of cardiomyocites, where the SR organelle, source of calcium ions, is in close proximity to the cell membrane. In fact the specific implementation is adapted from an earlier study by the same authors [1], where the electro-chemical coupling of two cardiomyocites was considered. Here instead of considering two distinct intracellular spaces and membranes separated by a thin extracellular space, the domain consists of single intra- and extracellular spaces connected via a thin membrane with embedded ionic channels. I think the manuscript will be of interest for the readership of PLOS Computational Biology and thus recommend its publication. It clearly fits within the journal scope, plus it is generally well-written and easy to follow (with detailed descriptions of boundary conditions and of how to deal with ionic channels). It will be accessible to a wide audience of computational biologists and numerical analysts looking for applications. However, several aspects need to be clarified and polished: main message, figures, physiological applications, etc. In its current form, the paper looks like a collection of numerical experiments lacking a clear conducting thread. I hope the suggestions below well help the authors improving the manuscript and its message.

Authors' response:

Thank you for these introductory remarks. It is, as you point out, difficult to escape the fact that the paper presents a collection of numerical experiments. However, the aim has been to use these experiments to clarify specific biophysical mechanisms involved in the action potential and calcium handling at the dyad. We appreciate your comment and will keep this criticism in mind as we work to improve the focus and coherence of the manuscript.

• **Dyadic space.** It is unclear how the electro-diffusion model presented here specifically applies to the dyadic space of cardiomyocites. Fig. 8 suggests that the rightward boundary of the intracellular domain would correspond to the sarcoplasmic reticulum membrane, but my understanding is that SR feedback and influence are not considered. As it is, the model could well describe generic ionic electro-diffusion dynamics near membrane-bound channels of subcellular domains of all types.

- I see that numerical experiments specific to the cardiac dyad are presented in section 3.9 (ryanodine receptors activation time, dyad width, . . .), but these should be presented early on I think.

Authors' response:

We see your point that, in our current setup, the SR membrane effectively acts as just another boundary. We also recognize that several preliminary experiments in the paper focus on the effects of individual ion channels and exchangers before addressing the question of how quickly calcium waves traverse the dyad. However, this organization was chosen deliberately as a step-by-step approach to build understanding of the underlying dynamics before turning to the specific question of dyadic calcium signaling.

That said, to avoid losing the reader on the way to the central results, we have revised the introduction to the *Results* section to more clearly indicate where the reader is headed and how the early experiments contribute to the final conclusions.

Text included in the new version:
In this section, we present the results of simulations using the model described above. The central goal is to investigate the electrodiffusive dynamics in the dyadic space of cardiomyocytes and to examine whether electrical effects, in addition to diffusion, significantly influence Ca^{2+} transport across the dyad.

The key quantity of interest is the time required for Ca^{2+} ions, entering through membrane channels, to reach the opposing RyRs on the SR membrane. However, before addressing this, we start with two simplified examples that illustrate general properties of the PNP system, such as the rapid decay towards electroneutrality and the formation of the Debye layer. These initial examples provide insights of the dynamics that also govern the dyadic space.

Next, we examine the dyad-specific simulations, including scenarios where different ion channels and exchangers are opened. We analyze how the diffusion coefficient and dyad width affect local ionic concentrations and the Ca^{2+} arrival time at the SR membrane. Finally, we compare the PNP model predictions with those of alternative modeling approaches, including reaction-diffusion equations and ODE-based compartment models, to clarify when the full PNP system is required.

• **Membrane curvature.** Can the numerical methodology presented here handle curved boundaries? Would that be relevant for dyadic space, where the SR structure presents highly irregular shape and morphologies?

Authors' response:
We have chosen to use MATLAB and finite differences to solve the PNP equations, as this approach gives us full control over how the equations are implemented and, in particular, how the membrane channels and exchangers are handled. This aspect is quite technical and not straightforward to get right. Handling more realistic geometries, including curved membranes, is certainly of interest and has been explored by others, such as Hake et al. (2012). However, such geometries are best addressed using finite element methods (FEM) and tools like, e.g., FEniCS or MFEM.

In our experience, these FEM-based tools provide less direct control over the implementation of membrane conditions, which are central to the questions we address here. For this reason, we have deliberately restricted ourselves to simple geometries, finite differences, and MATLAB for the current work. We have also implemented PNP solvers in MFEM using finite elements, but we are less confident in that code at this stage. We should emphasize that this lack of trust reflects our own limited experience with FEM programming, not any deficiency in the FEM approach itself, nor in the MFEM library as such.

• **Figures.** Would it be possible to provide more concise and synthetic figures, that would be more attractive to the reader? It's fine to have a short accompanying caption with the figure, but where is the need to add lengthy descriptions of each subplot and subfigure as in

Fig. 4 (A:, B:, C:, . . .) ? I feel many figures also contain redundant information, and the authors could focus on one ionic species density, with only one solution snapshot.

Authors' response:
We originally included Figure 4 in Section 2.6 to explain the general setup used in Figures 7, 9, 10, 11, 12, and 13. The idea was that explaining the figure organization once would make the subsequent figures easier to interpret. However, we now realize that placing Figure 4 in the Methods section while the related figures appear in the Results section is not ideal. We have therefore moved Figure 4 into the Results section (the figure is renumbered Figure 6 in the revised version).

Regarding the complexity of Figures 7, 9, 10, 11, 12, and 13, we agree that they are challenging to read, but we believe they present essential information for understanding the electrodiffusive dynamics in the dyad. If only diffusion were considered, it would be sufficient to show only the perturbed ion concentration (since the other ion concentrations would be left unchanged). However, when electrical forces are included, perturbations in one ion species affect the distributions of all the others through electrical interactions. For this reason, we find it necessary to display all four ion concentrations.

We considered reducing the number of time snapshots shown, but this would make the dynamics harder to follow. Many alternative plotting styles were tested before we settled on the current form, which we believe offers the best balance between completeness and readability.

• **Buffers and permanent charges.** One difference with the earlier model [1] is the inclusion of calcium buffers. Although their role and importance are clear from a modeling point of view, can the authors comment (and quantify) the influence of buffers on PNP numerical solutions? What differences do we see with and without buffers? Same question for the permanent charge density ρ from Eq. (8).

Authors' response:
We have now included a new section in the Supplementary Information (section S1) where we compare the solutions with and without calcium buffers. The permanent charge density is necessary in order to achieve physiological electroneutrality away from the membrane. We have included a comment about this in the revised manuscript (see Section 2.3).

• **Diffusion model.** Some conclusions about the pure diffusion model formation seems obvious to me. . . Of course by neglecting the electric field (section 3.10), no Debye layer near the membrane can be formed. It becomes a bit more interesting at the end of page 28, where the authors provide a reference on how to account for the voltage in a pure diffusion model. What is again the purpose of this section (3.10)? This remains unclear to me.

Authors' response:
The purpose of Section 3.10 (new Section 3.11) is to compare the solution of the PNP model to the solution of the simpler and more widely used reaction-diffusion model. Some aspects

of this comparison might, like the reviewer points out, be obvious for some readers. However, we believe that pointing out these differences is of relevance in this paper in order to get an impression of the differences between the two models. Moreover, the conclusion that the time of arrival dynamics for the calcium concentration is more or less identical in the PNP and the pure reaction-diffusion model did not seem obvious to us. In the new version of the manuscript, we have extended the investigation into the reason for this in Section 3.11 based on the comment from another reviewer. It basically turns out that the reason why these models appear to provide very similar solutions, is that the Ca-concentration in the dyad is very low effectively removing the electrical terms defining the difference between the models (see the new version, Section 3.11, for details).

Reviewer #3:

This manuscript presents a computational investigation into the electrodiffusion dynamics of ions within the cardiac dyad, employing the Poisson-Nernst-Planck (PNP) system of equations. The authors developed a three-dimensional computational model based on the PNP equations, simulating the concentrations of sodium (Na+), potassium (K+), calcium (Ca2+), and chloride (Cl−) ions, along with the electrical potential (φ). The model geometry consists of a simplified rectangular representation of the dyadic cleft (intracellular space), a segment of the adjacent cell membrane incorporating single, deterministically gated K+, Na+, and Ca2+ channels and the Na+/Ca2+ exchanger (NCX), and a portion of the T-tubule (extracellular space). The model also includes two types of stationary intracellular Ca2+ buffers, acknowledging their significant role in modulating free Ca2+ concentration. The PNP system is solved numerically using a finite difference method on an adaptive mesh, employing operator splitting to handle buffering reactions and an implicit time-stepping scheme for the coupled electrodiffusion equations.

The study reports several key findings. Simulations demonstrate the formation of a Debye layer—a region of non-electroneutrality—near the cell membrane at rest, emphasizing the contribution of electrical forces even in establishing equilibrium within the dyad. The results suggest that interactions between different ion species within the dyad are primarily mediated by electrical forces, an effect inherently missed by models based solely on diffusion. A sensitivity analysis reveals that the local Ca2+ concentration profile and the time required for Ca2+ ions released near the membrane to reach the vicinity of ryanodine receptors (RyRs) on the SR (used as a proxy for RyR activation time) are significantly dependent on the assumed width of the dyadic cleft and the intracellular Ca2+ diffusion coefficient (DCa). Furthermore, the simulations explore a scenario where localized Na+ accumulation near an open Na+ channel could potentially drive the NCX to operate in reverse mode sufficiently to trigger RyR opening, even without direct Ca2+ influx through L-type calcium channels (LTCCs). Comparisons with simpler models indicate that while the PNP model predicts faster relaxation towards electroneutrality compared to pure diffusion, a reaction-diffusion model yields similar estimates for the Ca2+ arrival time at RyRs, despite differences in the predicted ion concentrations very close to the membrane. ODE-based compartmental models failed to reproduce the sensitivity to dyad width and DCa observed in the PNP simulations.

The authors conclude that the necessity of employing the full PNP system for modeling the cardiac dyad depends critically on the specific scientific question being addressed. They propose that PNP is essential when investigating phenomena directly related to the Debye

layer or electrical interactions between different ion species. However, for estimating the timing of Ca2+ signal propagation across the dyad to the RyRs, simpler reaction-diffusion models might provide adequate accuracy

The findings of this paper offer potentially useful perspectives on the interplay between diffusive transport and electrical forces within this critical signaling domain. The novelty rests primarily on the specific application and the comparative aspect, rather than on methodological innovation. The work is a valuable contribution which applies a fundamental electrodiffusion model to the cardiac dyad and performs comparative analyses.

Nonetheless, the paper could benefit from a revisions according to the points below, in order to rigorously address its limitations, particularly the highly simplified geometry used, the deterministic gating for single ion channels which do not take into account the known structural complexity and stochastic behavior of the dyad, and the known limitations of the standard PNP formalism itself, particularly concerning its applicability in nanoscale environments where effects like finite ion size and ion-ion correlations, neglected in the standard model, can become prominent.

Authors' response:
Thank you for these thoughtful general remarks.

Major Points:

1. Justification and discussion of Standard PNP: The authors should provide a clear rationale for employing the standard PNP formulation in the nanoscale dyadic environment, explicitly addressing why potential limitations related to ion size (steric effects) and ion-ion correlations are considered negligible or acceptable for the study's goals; see e.g. Zheng, Qiong, and Guo-Wei Wei. "Poisson–Boltzmann–Nernst–Planck model." The Journal of chemical physics 134.19 (2011). This discussion should consider the potential impact of these neglected physical factors on the predicted concentration profiles, Debye layer structure, and electrical interactions. The limitations section should explicitly mention these standard PNP assumptions, and conclusions should be tempered accordingly.

Authors' response:
We agree that, although the PNP equations adequately incorporate electrical effects, important assumptions remain, as the reviewer points out. In particular, the standard PNP formulation does not account for finite ion sizes (steric effects) or ion-ion correlations.

In the revised manuscript, we have addressed the issue of ion crowding by implementing a version of the PNP equations that includes steric effects, following the approach of Lu et al. (2011); reference [56] in the revised version. This extension prevents unphysiological accumulation of ions in confined spaces. The results of these simulations and the corresponding model description are now presented in Section S6 in the Supplementary Information.

We note, however, that ion-ion correlations, in the sense of beyond mean-field statistical

interactions between individual ions, are not included in our model. These effects may be relevant in some nanoscale systems but are outside the scope of the current study.

The following text has been included in a new subsection of the discussion (Section 4.8):
Several more detailed modeling approaches exist that extend beyond the standard PNP formalism used here. These include modified electrodiffusion models that incorporate finite ion sizes (elaborate models of steric effects), ion-ion correlations, or additional corrections at the nanoscale, see, e.g., [56]. In the Supplementary Information (see Section S6 in S1 Appendix), we extend the PNP model described above to account for steric effects following the approach in [56]. However, we observe that including steric effects do not have a significant effect on the results under the conditions considered in this study.

2. Impact of geometry simplifications: The manuscript could benefit from an expanded discussion on the consequences of using a simplified rectangular geometry and deterministic single-channel gating. How might realistic, irregular dyad morphologies and the stochastic, clustered nature of LTCC and RyR gating (see e.g. Jones, Peter P., Niall MacQuaide, and William E. Louch. "Dyadic plasticity in cardiomyocytes." Frontiers in Physiology 9 (2018): 1773) alter the reported findings concerning Debye layers, cross-species electrical interactions, sensitivity to parameters, and the NCX-triggering scenario?

Authors' response:
The simplified rectangular geometry and deterministic single-channel gating adopted in our model certainly abstract away much of the structural complexity and stochastic channel behavior that are known features of the cardiac dyad. Complex geometry, stochastic opening and closing of channels, and electrodiffusive processes are all essential characteristics of dyadic dynamics. Here, we focus specifically on the electrodiffusion aspect.

We could have included stochastic channel gating, but for clarity, we chose to concentrate on the simpler case of channels being forced to be either open or closed. As also explained in our response to another reviewer, we are not able to handle complex geometries with our current tools — finite differences and MATLAB. Handling more realistic geometries would require the use of finite element methods and tools like FEniCS or MFEM (or similar). Given the complexities of the equations, particularly the treatment of ion channels and exchangers, we have deliberately chosen to work with methods that provide us with full control of the numerical implementation.

We fully acknowledge the limitations pointed out by the reviewer and now discuss them explicitly in the revised Discussion section (Section 4.5). In particular, we note that more realistic reconstructions of dyadic geometry, such as the tomographic models presented by Hake et al. (2012), show that the dyadic cleft and SR membrane surfaces can be highly irregular. This irregularity may lead to spatial heterogeneity in ionic concentrations and electric fields that are not captured by our simplified geometry. Similarly, as reviewed by Jones et al. (2018), RyR and LTCC channels often occur in clusters, and their gating exhibits

stochastic behavior and possible coupled gating phenomena. In contrast, our model assumes deterministic gating of single channels.

We stress, however, that the aim of the present work has been to isolate and understand the fundamental consequences of electrical interactions within a controlled and simplified setup. This choice allows us to examine the role of electrodiffusion directly, without the added complexity of geometry and gating stochasticity.

Text included in the new version (Section 4.5):
In addition, the present model uses a simplified rectangular geometry for the dyadic space and assumes deterministic gating of single ion channels. These choices abstract away much of the structural complexity and stochastic channel behavior that are known features of the cardiac dyad. In particular, detailed reconstructions [30, 52] have shown that the surfaces of the dyadic cleft and the SR membrane can be highly irregular, potentially leading to spatial heterogeneities in ion concentrations and electric fields that are not captured by the present approach. Also, ion channels in physiological membranes often appear in clusters rather than as isolated entities, see, e.g., [53, 54], but for simplicity, we have used only one channel of each type in our simulations. This entails one Na^+ channel per 1 μm^2, tuned to reproduce a realistic upstroke velocity within the modeled domain. Furthermore, experimental studies and reviews [55] have demonstrated that clusters of L-type Ca^{2+} channels and RyRs display stochastic gating behavior and possible coupled gating phenomena. Our model does not include these aspects, as we chose to focus on the electrodiffusion dynamics under the assumption of deterministic channel behavior. This was done to isolate and examine the fundamental consequences of electrical forces on ionic transport without the additional complexity of geometry and gating stochasticity.

3. Deeper interpretation of PNP vs. RD comparison: The observation that PNP and RD yield similar Ca2+ arrival times could benefit from a deeper analysis. The authors should investigate and discuss the reasons behind this similarity. Does it imply electrical forces are truly negligible for bulk transport across the cleft under these conditions, or are other factors at play? This analysis is crucial for substantiating the conclusion about the conditional sufficiency of RD models.

Authors' response:
Thank you for this comment encouraging us to investigate this similarity more thoroughly. We have now included a new figure in the manuscript (Figure 18) which displays the size of the different terms in the equation governing the calcium concentration. In these plots, we observe that under these conditions, the electrical term is more or less negligible.

The following the following text has been included added to the Results (Section 3.11.3):
In Figure 18, we investigate the similarity between the PNP model and the pure reaction-diffusion model by comparing the size of the three terms in (6) for $k = Ca^{2+}$. We consider points at different distances, d, from the intracellular mouth of the Ca^{2+} channel. We observe that the diffusion term, B_d, is by far the largest term in the equation. The small magnitude of the electrical term, B_e, compared to the diffusion term, B_d, explains why the difference between the PNP and the pure reaction-diffusion model is so small. In the

Supplementary Information (see Figure S7 in S1 Appendix), we similarly compare the size of the different terms for the Na^+ concentration following the opening of the Na^+ channel. We observe that for Na^+, the size of B_e is more comparable to the size of B_d, and there is a more prominent difference between solutions of the PNP model and the pure reaction-diffusion model. Since the intracellular Na^+ concentration is about 1000 times larger than the intracellular Ca^{2+} concentration in these simulations, this suggests that the negligible impact of the electrical term for the Ca^{2+} concentration might be related to the small intracellular Ca^{2+} concentration.

In addition, the following the following text has been included added to the Discussion (Section 4.2):
In the simulations of intracellular dynamics following the opening of a membrane Ca^{2+} channel, however, we observe that the electrical term, B_e, is almost negligible compared to the diffusion term, B_d (see Figure 18). As a result, the pure reaction-diffusion equation was able to reproduce the time of arrival estimates for Ca^{2+} at the RyR computed using the full PNP model (see Figure 17). The small magnitude of the B_e term in the dyad likely reflects the low Ca^{2+} concentration in that region (cf. Section S4 in S1 Appendix).

4. Contextualization with advanced models: The discussion should briefly acknowledge the existence of more sophisticated electrodiffusion models (e.g., PNPF, PNPB, steric PNP) and advanced stochastic dyad models to provide better context for the current work's position, scope, and limitations within the broader field of computational dyad modeling (see e.g. Colman, Michael A., et al. "Multi-scale computational modeling of spatial calcium handling from nanodomain to swhole-heart: Overview and perspectives." Frontiers in Physiology 13 (2022): 836622).

Authors' response:
We have now included a new subsection in the Discussion addressing more advanced electrodiffusion models (see Section 4.7). In addition, we address stochastic dyad models in Section 4.6 (see answer to comment 2 above).

4.8 More advanced computational models of electrodiffusion dynamics
Several more detailed modeling approaches exist that extend beyond the standard PNP formalism used here. These include modified electrodiffusion models that incorporate finite ion sizes (elaborate models of steric effects), ion-ion correlations, or additional corrections at the nanoscale, see, e.g., [56]. In the Supplementary Information (see Section S6 in S1 Appendix), we extend the PNP model described above to account for steric effects following the approach in [56]. However, we observe that including steric effects do not have a significant effect on the results under the conditions considered in this study.

Alternative approaches to modeling electrodiffusion at the nanometer scale include models that describe ion transport directly within the channel structures themselves, using PNP equations with steric effects or related formalisms, see [57]. These models address ion interactions and crowding within the confined environment of the channel pore, representing a more accurate and elaborate modeling strategy to the approach taken here.

5. Numerical validation evidence: In order to bolster confidence in the simulation results, it would be interesting to provide evidence of numerical convergence (e.g., mesh refinement study showing convergence of key outputs) and code validation (e.g., comparison with analytical solutions for simplified test cases, if possible), either in the main text or supplementary materials.

Authors' response:
In the supplementary materials (see Figure S3 and Figure S4), we have now added a convergence analysis of the computations presented in Figure 4 and Figure 12. Specifically, we show solutions of the problem for several spatial resolutions and time step sizes. Convergence is assessed by direct visual comparison of the solution curves for the ionic concentrations. The results indicate reasonable convergence with respect to both spatial discretization and time stepping. A reference to these results is now included in the main text.

To our knowledge, analytical solutions of the PNP system exist only in stationary, highly simplified settings, and typically require linearization of the exponential nonlinearity. We have implemented such a linearized version and obtained results that compare reasonably with our numerical solutions. However, we do not consider this a meaningful validation for the full nonlinear, time-dependent problem considered here, and therefore do not include these comparisons in the main text.

Minor Points:

1. Boundary condition justification: Is it possible to provide a more explicit physiological justification for the choice of boundary conditions, particularly the no-flux Neumann conditions for concentrations at the domain edges?

Authors' response:
In the revision, we have clarified the formulation of the boundary conditions, which were not fully stated in the original version. In particular, we now emphasize that the Neumann boundary conditions imply that the ionic fluxes across the boundary are zero, not that the normal derivative of the concentrations is zero.

The only boundary close to the considered dynamics is the boundary on the right-hand side of the dyad (representing the SR membrane). Since this membrane is assumed to be closed for diffusion of ions, a no-flux boundary condition for the ionic concentrations was deemed to be reasonable at this location. Furthermore, the remaining boundaries are located far from the ion channels and are not assumed to have a significant effect the considered dynamics. Dirichlet boundary conditions could have been used on most of these boundaries instead of Neumann boundary conditions, but to achieve a reasonable Debye layer, Dirichlet boundary conditions cannot be applied on the boundaries close to the membrane. For simplicity we therefore use Neumann boundary conditions on all boundaries.

The following text has been added to Section 2.2.1:
These boundary conditions amount to no flow of ions across the domain boundary. At the rightmost boundary this represents that the membrane of the SR is closed for flow of ions. The remaining boundaries are located quite far from the ion channels and the considered dynamics and Neumann boundary conditions are chosen for simplicity and because they, unlike Dirichlet boundary conditions, do not interfere with the formation of the Debye layer when applied close to the membrane.

2. The bottom panels of Figs. 4, 7, 9 – 13 show two curves of the temporal evolution of the solutions in two spatial points. The location of these points is described in words in sec. 2.6 but it is a bit complex. It would be better for the reader to mark these two points in a figure, for example in Fig. 3 or in the first top-left panel of Fig. 4.

Authors' response:
We see the point raised here and have now added the locations of these points directly to the figure used to explain the setup of the simulations. This figure is now numbered Figure 6 and has been moved to the Results section, following a suggestion from another reviewer.

3. The computing time for Figures 9 -13 was 6 hours total or for each figure?

Authors' response:
It was the computing time for each figure. This is now clarified in the text.

4. Regarding iterative solvers for the EMI model, in addition to [19] it would be more comprehensive to also cite the recent work by Huynh, Ngoc Mai Monica, et al. "Convergence analysis of BDDC preconditioners for composite DG discretizations of the cardiac cell-by-cell model," SIAM Journal on Scientific Computing 45.6 (2023): A2836-A2857.

Authors' response:
The paper by Huynh et al. is now cited alongside the other references concerning subcellular models.
